# Late-gestation heat stress in Holstein dams programs in utero development of daughter's germline, triggering skin and hair morphology adaptations of granddaughters

**DOI:** 10.3168/jdsc.2023-0400

**Published:** 2023-08-19

**Authors:** B.D. Davidson, E.T. Gonzales, G.L. Mast, J. Laporta

**Affiliations:** Department of Animal and Dairy Sciences, University of Wisconsin–Madison, Madison, WI 53706

## Abstract

Homeostasis and thermoregulation are influenced by the interplay of hair coat and skin characteristics. Our previous work indicated that hair and skin adaptations, triggered by in utero heat stress, affect thermoregulation in postnatal life. Herein, we investigate multigenerational carry-over effects of late-gestation heat stress on hair and skin characteristics beyond the first generation. Pregnant Holstein dams (F_0_, grand-dams) were heat stressed (HT, shade, n = 41) or provided active cooling (CL, shade, fans, and water soakers, n = 41) for the last 56 d of gestation during summer (temperature-humidity index ≥68). The first generation of heifers (F_1,_ daughters) who were in utero heat stressed (HT_F1_, n = 36) or not (CL_F1_, n = 37) were born and raised as a cohort from birth to first calving. Thirty second-generation heifers (F_2_, granddaughters), born to HT_F1_ (HT_F2_, n = 12) and CL_F1_ (CL_F2_, n = 18), were raised as a cohort from birth to 70 d of age. Hair samples and skin biopsies from HT_F2_ and CL_F2_ were collected on postnatal d 70 (n = 6/group). Hair samples were subdivided into topcoat and undercoat, and skin tissue was fixed for ~18 h in 10% formalin, dehydrated, paraffin-embedded, sectioned, and stained with hematoxylin and eosin to visualize morphology. Variables analyzed included hair length and diameter; stratum corneum cross-sectional area and thickness; epidermis thickness; sweat gland depth, number, cross-sectional area, and average size; and sebaceous gland number, cross-sectional area, and average size. Measurements were performed using the ImageJ software and analyzed using PROC MIXED in SAS (SAS Institute Inc.). Hair length and diameter tended to be shorter and thicker in HT_F2_, compared with CL_F2_. The HT_F2_ skin had smaller stratum corneum cross-sectional area and tended to a thinner epidermis. to CL_F2_, HT_F2_ skin had more but smaller sebaceous glands, whereas no differences in sweat glands were observed. In summary, we report phenotypic alterations in hair and skin characteristics of granddaughters. Whether these adaptations grant improved postnatal thermoregulatory ability for the granddaughters remains to be investigated.

## Highlights

• Late-gestation heat stress affects the gestating calf and its developing germline.
• Heifers from heat-stressed germlines had shorter and thicker hair and thinner skin.
• Heifers arising from heat-stressed germlines had more but smaller sebaceous glands.

## Graphical Abstract

**Figure:**
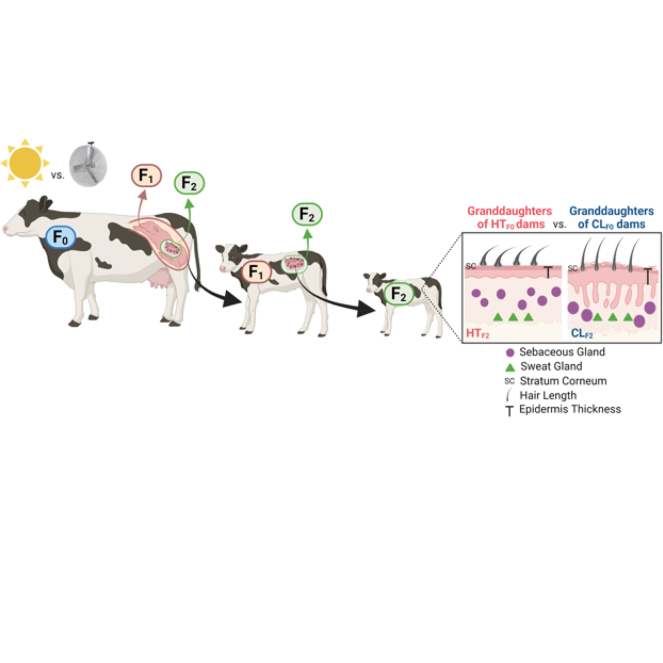
**Summary:** During times of high temperature and humidity, an animal's hair, coat, and skin play essential roles in heat dissipation and thermoregulation. Late-gestation heat stress, experienced by the pregnant dam (F_0_ generation), has been shown to alter her daughter's (F_1_ generation) hair length and distribution of sebaceous and sweat glands through the first year of life. The germline (F_2_), which will give rise to the second generation, is indirectly exposed to the intrauterine heat-stress insult of the developing fetus (F_1_). Herein, we report a multigenerational effect, whereby the F_2_ generation exposed to in utero heat stress as a germ cell has shorter and thicker hair, decreased stratum corneum area and epidermis thickness, and more numerous but smaller-sized sebaceous glands at 70 days postnatal. These alterations in hair and skin characteristics of the heat-stressed germline may grant the second generation (F_2_) improved thermoregulatory abilities in their postnatal lives.

The integumentary system comprises the epidermis, dermis, hypodermis, and appendageal structures that consist of sebaceous glands (**SBG**), sweat glands (**SWG**), and hair follicles. The initiation of hair follicle growth in *Bos taurus* breeds begins in utero as early as 77 d of gestation, and hairs begin emerging by 203 d (Lyne and Heideman, 1959). While initiation begins early in gestation, maturation is a gradual and dynamic process. For instance, at 7 to 8 mo of gestation, skin layers appear more distinct, hair follicles are more prominent, and SBG and SWG are lobed and elongated, compared with skin structures at 6 to 7 mo of gestation (Al-Salman et al., 2020). Disturbances of the intrauterine environment during conception and gestation can alter offspring phenotypes through adulthood (Barker, 1990; Desai and Hales, 1997). Optimal maturation of skin layers, skin glands, and hair is important to achieve postnatal thermal homeostasis and adequate protection from environmental influences.

To maintain core body temperatures within a normal physiological range, cattle balance heat loss and heat gain through sensible and latent heat transfer routes (Hahn, 1999). As the heat load accumulates, cattle become less effective at maintaining core body temperatures and might succumb to heat stress (Bernabucci et al., 2010). The US dairy industry loses an estimated $1.4 billion annually from lower milk production and reduced productive life from cows under heat stress in their dry period (Ferreira et al., 2016) and from their daughters (first generation) who experienced in utero heat stress (Laporta et al., 2020). The negative effects of maternal late-gestation heat stress on health (Tao et al., 2012; Dado-Senn et al., 2020), growth, and organ development of the resulting daughters (Monteiro et al., 2014; Dado-Senn et al., 2021) have been documented. Moreover, Holstein heifers that experienced in utero heat stress have altered hair and skin characteristics at birth, which remain at 1 yr of age (Davidson et al., 2022). Specifically, in utero heat-stressed daughters had more numerous but smaller-sized SBG, longer hair, fewer and smaller-sized SWG, and reduced SWG coverage in the skin layers.

An area of growing interest is the effect of in utero insults on the programming of the developing fetuses' germline that will give rise to the second generation, passing on genetic and epigenetic information (Houston et al., 2018; Laporta et al., 2020; Yadav et al., 2022). The germline is essential to a population's survival and must sense potential stressors to trigger adaptive and protective mechanisms to maintain cell quality and regulate gene expression (Latham, 2016; Schisa, 2019). Although substantial reductions in milk production and survival of the second generation, arising from in utero heat-stressed germ cells, have been reported (Laporta et al., 2020), alterations of hair and skin properties have not been documented. Herein, we investigated whether fetal germline exposure to intrauterine hyperthermia triggers hair and skin adaptations in the resulting granddaughter.

The Institutional Animal Care and Use Committees at the University of Florida (protocol #201910599) and the University of Wisconsin–Madison (protocol #A006415-A03 and #A006602) approved this longitudinal multigenerational study conducted from August 2020 to October 2022. The experimental design and dam treatments are described by Dado-Senn et al. (2021). Briefly, for the last 56 d of gestation, pregnant Holstein dams (**F_0_**, grand-dam, n = 82) were exposed to heat stress (**HT_F0_**, shade, n = 41) or provided heat stress abatement via active cooling (**CL_F0_**, shade, fans, and water soakers, n = 41) at a commercial farm in Trenton, Florida, during the summer of 2020. The temperature and relative humidity equation proposed for subtropical environments (NRC, 1971; Dikmen and Hansen, 2009) was used to calculate the temperature-humidity index (**THI**). The hourly THI remained above 68, indicating that all grand-dams (F_0_) experienced environmental heat stress during late gestation. However, grand-dams with access to heat abatement via active cooling had reduced thermal indices, indicating thermoneutrality was achieved (Dado-Senn et al., 2021).

Offspring born to F_0_ grand-dams (**F_1_**, daughters, n = 73) experienced in utero heat stress (**HT_F1_**, n = 36) or not (**CL_F1_**, n = 37). The daughters were managed as a single cohort from birth until first calving and were not experimentally exposed to heat stress or heat stress abatement during this period (Dado-Senn et al., 2021; Davidson et al., 2022). At approximately 13 mo of age, the daughters were artificially inseminated at the University of Wisconsin–Madison Marshfield Agricultural Research Station. Approximately 2 mo before calving, the daughters were transported to the Arlington Dairy Research Center, where they gave birth to the granddaughters (**F_2_**, n = 30) in the fall 2022. Thus, the germlines, resulting in F_2_ granddaughters, were exposed to in utero heat stress (**HT_F2_**, n = 12) or not (**CL_F2_**, n = 18) through the fetal daughter (F_1_) during late gestation. The granddaughters were raised in individual sand-bedded polyethylene calf hutches (Calf-Tel, L. T. Hampel Corp.) and managed as a single cohort according to the standard operating procedures of the Arlington Dairy Research Center. Milk weaning began at 42 d of age and was complete by 49 d. At 56 d of age, groups of 4 calves were moved to group calf hutches (Calf-Tel, L. T. Hampel Corp.).

At 70 d of age, hair samples and skin tissue biopsies were collected from the right side of the neck (within the injection triangle), from a subset of F_2_ heifers (n = 6/group). Hair was stored in pre-labeled plastic bags at room temperature until further analysis. Skin tissue was harvested with a sterile biopsy punch (Standard Biopsy Punch, 6 mm, Integra Miltex Life Sciences Corporation, York, PA). Biopsied tissue was rinsed in sterile PBS, fixed at room temperature in 10% neutral-buffered formalin for 16 to 24 h, bisected, placed in histology cassettes, and stored in PBS at 4°C. To visualize skin morphology, tissue was dehydrated, paraffin-embedded, sectioned (7 μm), fixed to glass slides, and stained with hematoxylin and eosin (**H&E**; Hematoxylin 7211, Clarifier1, Bluing, and Eosin Y Alcoholic; Thermo Fisher Scientific). Hair and skin tissue morphological measures were performed using the ImageJ software (US National Institutes of Health, Bethesda, MD) following procedures described by Sarlo Davila et al. (2019) and Davidson et al. (2022). Succinctly, hair was initially categorized into short or long lengths (undercoat and topcoat, respectively). Ten hairs of each length were measured to average the length of all hairs, average of short hairs, and average of long hairs. The difference between the undercoat length and topcoat length was calculated and recorded. Hair diameter was measured and averages were calculated for all hairs, undercoat, and topcoat (Figure 1). The H&E-stained skin slides were imaged using the Keyence BZ-X800 (Keyence Corporation, Japan) microscope at 40× (10× lens magnifier and 4× objective lens) and cropped to 1,000 × 1,000 pixels. Measurements of interest included stratum corneum (**SC**) cross-sectional area and thickness; epidermis thickness; SWG depth, number, cross-sectional area, and average size; and SBG number, cross-sectional area, and average size (Figure 1).

**Figure 1:**
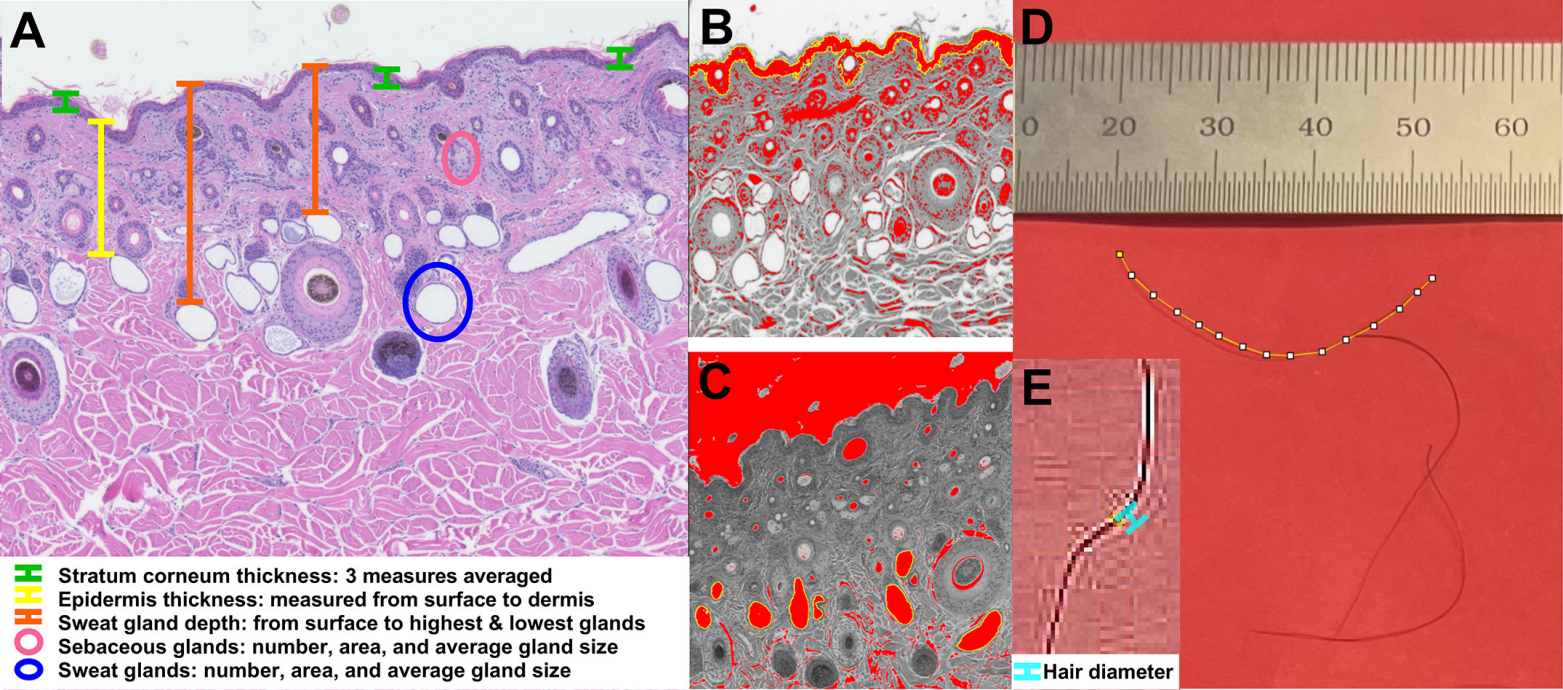
Diagram of measurements obtained from the skin tissue biopsies and hair samples. For skin tissue, the measurements of interest included stratum corneum thickness (A; green lines), epidermis thickness (A; yellow line), sweat gland depth (A; orange lines, proximity to skin surface), sebaceous gland number and cross-sectional area (A; pink circle), and sweat gland number and cross-sectional area (A; blue circle). Average sebaceous gland and sweat gland sizes were calculated by dividing the total cross-sectional area by the number of glands counted in each image. The stratum corneum cross-sectional area was measured utilizing thresholding, whereby the software identified darker image pixels in the skin surface layer (seen in red) and manually selected for measurement (B). The sebaceous gland cross-sectional area was measured using a freehand tracing tool, and the sweat gland cross-sectional area was quantified by thresholding (i.e., lighter pixels within the glands were identified). The threshold-altered photos were cross-referenced against the original photo to be sure only sweat glands were selected for measurement (C). Hair samples were visually assessed, divided into short and long, and individually measured using the segmented line tool. The images were sharpened (increasing contrast and detail) for hair diameter measurement.

Statistical analyses were performed in SAS (version 9.4, SAS Institute Inc., Cary, NC). Data were tested and evaluated for covariance and normality using Levene's test and Shapiro-Wilk statistic (Univariate procedure, SAS). Hair and skin measurements (i.e., hair length and diameter; SC cross-sectional area and thickness; epidermis thickness; SWG depth, number, cross-sectional area, and average size; and SBG number, cross-sectional area, and average size) were analyzed, utilizing a generalized linear mixed model. The statistical model included the main effect of the environmental treatment the grand-dam was exposed to in late gestation. Significance was declared at *P*-value ≤0.05 and tendencies at 0.10 ≥ *P*-value >0.05. Data are presented as least squares means ± standard error.

At 70 d of age, the length of the undercoat and the length difference between the undercoat and the topcoat were not different between treatment groups (*P* ≥ 0.12; Table 1). Yet, the average length of all hairs (both undercoat and topcoat) and the topcoat length tended to differ between groups. More specifically, HT_F2_ heifers had overall shorter hairs (*P* = 0.08) and had shorter topcoats (long hairs, *P* = 0.06), relative to CL_F2_ heifers (Table 1). Additionally, hair diameter was greater in HT_F2_ heifers: the average diameter of all hairs (combined undercoat and topcoat), as well as the undercoat diameter, and the topcoat diameter were thicker in the HT_F2_ heifers compared with CL_F2_ (all *P* ≤ 0.05; Table 1).

**Table 1:** Hair length and diameter of granddaughters at 70 d postnatal

Variable	Group^1^		SEM	*P*-value (grand-dam treatment)^1^
	Granddaughters of CL_F0_ dams (CL_F2_, n = 6)	Granddaughters of HT_F0_ dams (HT_F2_, n = 6)		
Hair length^2^				
Avg. length	19.56	15.67	1.37	0.08
Avg. short hair	12.91	10.74	1.27	0.26
Avg. long hair	26.21	20.60	1.84	0.06
Dif. S&L	13.30	9.87	1.42	0.12
Hair diameter^2^				
Avg. width	0.29	0.35	0.01	0.002
Short hair width	0.28	0.34	0.01	0.004
Long hair width	0.29	0.37	0.01	0.003

1 Grand-dams were exposed to either late-gestation in utero heat stress (HT) or cooling (CL), therefore exposing the F_1_ generation and her germline to heat stress or not. The germlines gave rise to the granddaughters (F_2_ generation).

2 Hair was collected from the neck location of HT_F2_ and CL_F2_ at 70 d. Hair characteristics include Avg. length = average length of all short and long hairs; Avg. short hair = average length of only short hairs; Avg. long hair = average length of only long hairs; Dif. S&L = the difference between the length of the short hairs and the long hairs; Avg. width = average diameter of all short and long hairs; Short hair width = average diameter of only short hairs; and Long hair width = average diameter of only long hairs. All units are millimeters. Significance was declared at *P*-value ≤0.05 and tendencies at 0.10 ≥ *P*-value >0.05.

The SC cross-sectional area of the skin was smaller (39.54 vs. 53.41 ± 3.63 µm^2^, *P* = 0.02; HT_F2_ vs. CL_F2_, respectively; Figure 2C) and the epidermis thickness tended to be thinner (0.009 vs. 0.01 ± 0.0006 mm, *P* = 0.09; Figure 2D) in the HT_F2_ heifers, relative to CL_F2_. Stratum corneum thickness, SWG depth, SWG number, SWG cross-sectional area, SWG average size, and SBG cross-sectional area were not different (*P* ≥ 0.17). Meanwhile, SBG were more numerous (21 vs. 14 ± 2 glands, *P* = 0.05; Figure 2E) but SBG average size (0.81 vs. 1.31 ± 0.09 µm^2^, *P* = 0.004, Figure 2F) was smaller in the HT_F2_ heifers, compared with CL_F2_ heifers.

**Figure 2:**
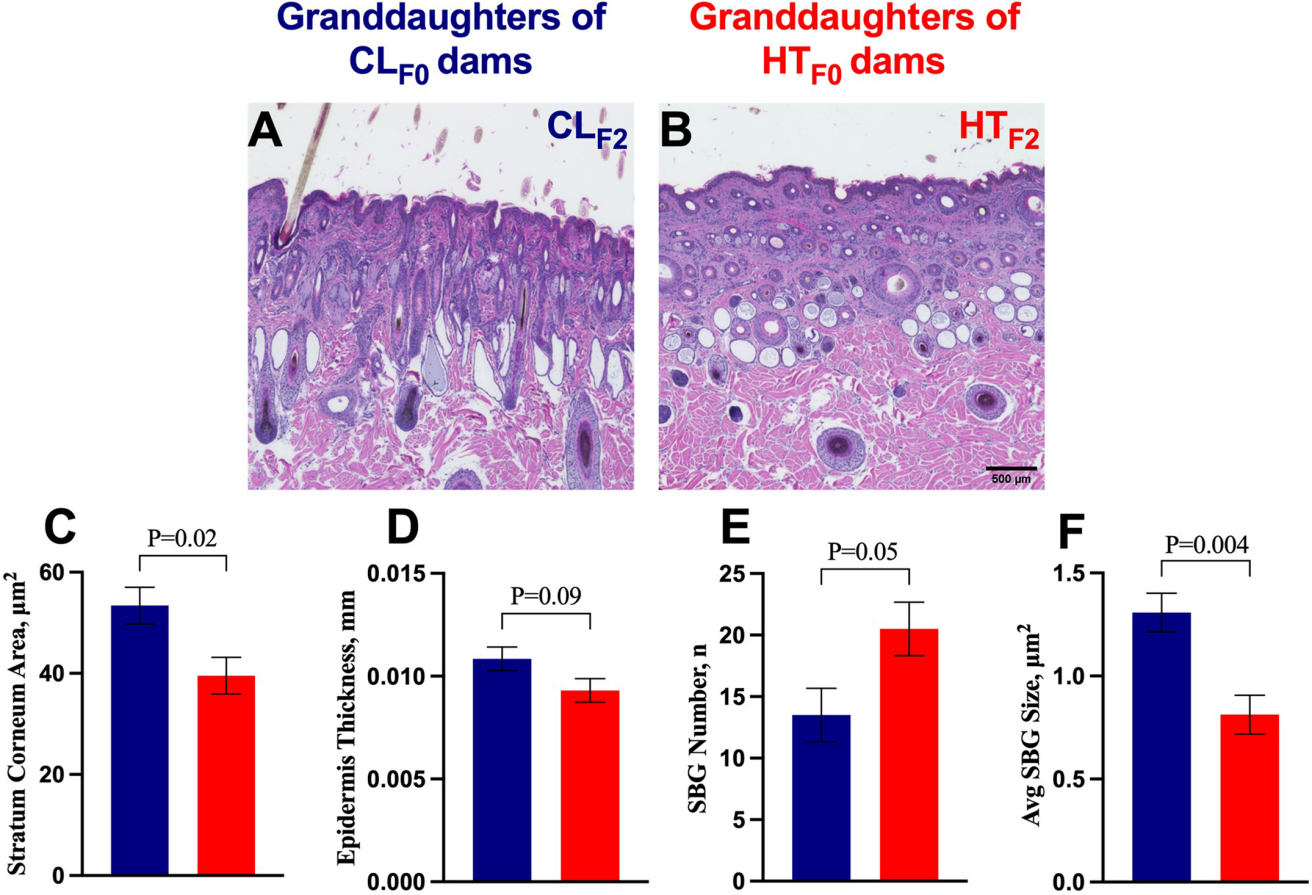
Skin histomorphology of granddaughters at 70 d postnatal. The germlines of the F_1_ generation, resulting in granddaughters, were exposed to in utero heat stress (HT) or not (CL) during late gestation. Skin tissue biopsies were collected from the neck of the granddaughters (F_2_ generation; HT_F2_ and CL_F2_, red and blue columns, respectively) at 70 d of age (n = 6/group) and stained with hematoxylin and eosin to visualize tissue architecture (A, B). Significantly different variables included stratum corneum cross-sectional area (C), epidermis thickness (D), sebaceous gland (SBG) number (E), and average (Avg) sebaceous gland size (F). Significance was declared at *P*-value ≤0.05 and tendencies at 0.10 ≥ *P-*value >0.05. Data are presented as LSM ± SE. Scale bar = 500 µm.

Lucas (1991) defines “programming” as the resulting long-term functional consequences of a physiological system by an early-life insult, and “the thrifty phenotype” was proposed by Hales and Barker (1992) to suggest that increased survivability, in like conditions, is an adaptive response to poor fetal environments. More recently, the idea of developmental plasticity has prompted numerous studies showing the environment in which one generation develops can affect the development of following generations (reviewed by McMillen and Robinson, 2005). Intrauterine heat stress insult to a developing fetus during late gestation has negative effects on the postnatal calf; including lower birth weight, compromised growth, and altered skin properties (Collier et al., 1982; Monteiro et al., 2014; Dado-Senn et al., 2021; Davidson et al., 2022). However, the extent to which late gestational heat stress affects the germline (i.e., the females' germline) of the developing bovine fetus has received less attention.

Studies in nematodes and plants suggest phenotypic responses to environmental conditions, experienced by the grandparent generation, can carry through multiple generations. For example, multiple generations of offspring from heat-shocked nematodes, *Caenorhabditis remanei*, are less equipped to survive severe heat shock or respond to changes in their environments (Sikkink et al., 2014). Moreover, a transgenerational effect (evidenced in the unexposed, third generation) is observed in *Arabidopsis thaliana* plants that are exposed to caterpillar herbivory and pathogens. Induced resistance to attack originates from a priming of acid-dependent defense, a response that persists for multiple generations (Rasmann et al., 2012; Luna et al., 2012). Hedhly et al. (2020) reports acute heat stress during vital developmental stages can lead to *A. thaliana* male sterility and can affect the ploidy of gametes.

Less is understood about the extent to which epigenetic inheritance of the ancestors' environment occurs in vertebrates. It has been documented that heat stress has direct effects on the development and quality of the bovine oocyte (Rocha et al., 1998; Rutledge et al., 1999; Al-Katanani et al., 2002; Roth and Hansen, 2004) and induces damage to the male germ cells in mice (Houston et al., 2018). In domesticated pigs, it has been shown that the second generation responds to a methyl-enriched diet fed to the grandparent generation (Braunschweig et al., 2012). Additionally, reductions in milk production and survival of the second generation, born from in utero heat-stressed Holstein dams, was reported by Laporta et al. (2020). Furthermore, a transgenerational effect was demonstrated in 12 production and reproduction traits of Israeli Holstein cows by Weller et al. (2021). These authors reported that production and calving traits are negatively affected in the second and third generations if the grand-dam was in the second half of pregnancy during hot summer months.

Our laboratory has previously documented in utero programming effects of hair and skin properties of Holstein heifers (Davidson et al., 2022) at birth, 2 mo, and 1 yr of age. Herein, we report long-term multigenerational phenotypic effects of hair and skin characteristics driven by in utero heat stress experienced by the fetal daughter (F_1_) and her developing germline (F_2_). This is the first experimental documentation of hair and skin adaptations in second-generation Holstein heifers.

In the present study, F_2_ heifers had overall shorter and thicker hairs. Interestingly, findings from the F_1_ generation in Davidson et al. (2022), show in utero heat-stressed heifers (HT_F1_) had overall longer hairs. Hair thickness was not analyzed in Davidson et al. (2022) but does play an important role in thermal tolerance (Collier and Gebremedhin, 2015). Yeates (1955) suggests that animals with long hair coats struggle to adapt to hot climates and might be more susceptible to heat stress. While thick hair is believed to trap sweat, thereby reducing the heat dissipation efficiency, the shorter hair coat observed in F_2_ heifers is similar to that of the Slick phenotype (Carvalho et al., 1995; Olson et al., 2003; Dikmen et al., 2014). Although speculative at this point, this adaptation might potentially grant them an advantage for thermoregulatory adaptivity. The F_1_ generation may program the second generation (F_2_) for heat stress tolerance, in preparation for hot climates.

In addition to the hair coat, the skin layers also play important roles in controlling body temperature and maintaining homeostasis (Ebling et al., 1992; Singh et al., 2013). Skin consists of the epidermis, dermis, and hypodermis. Within the epidermis (from deepest to most superficial) is the stratum basale, stratum spinosum, stratum granulosum, stratum lucidum, and SC. Many species, like the Windsnyer pig and the Indian buffalo, have thinner epidermis layers, supporting increased heat loss from the skin's surface (Saravanakumar and Thiagarajan, 1992; Moyo et al., 2018). In the present study, 70-d-old heifers had decreased SC cross-sectional area and thinner epidermis thickness. These findings are similar to results from the F_1_ generation (Davidson et al., 2022); however, the decreased SC cross-sectional area was only different at birth and by 2 mo of age SC cross-sectional area and epidermis thickness did not appear to have an impact on thermoregulatory ability. For the F_2_ generation to maintain smaller SC cross-sectional area and thinner epidermis thickness after 2 mo of age suggests an adaptation to multigenerational heat stress exposure.

Within these skin layers, each hair follicle is associated with SBG and SWG. To protect against skin damage and heat stress, SBG release sebum and SWG bring water to the skin's surface for evaporative heat loss (Saxena et al., 1994; Berman, 2011). In the present study, F_2_ heifers generated from late-gestation in utero heat-stressed germlines had smaller averaged sized but more numerous SBG with no differences in SWG number or size. These results are in agreement with the findings from birth in the F_1_ generation, whereby heat-stressed heifers had more but smaller averaged sized SBG (Davidson et al., 2022). However, by 2 mo of age the in utero heat-stressed heifers (F_1_) had fewer SBG and no differences in size were evident (Davidson et al., 2022). The roles of SBG are to prevent dehydration (Porter, 1993) and discourage sweat formation and loss from the skin's surface (Porter, 2001). In the current study, SBG function was not evaluated, but it is tempting to speculate that SBG, which are smaller and spread more numerously through the skin layers, may be programmed to protect the skin from drying out in high thermal conditions.

In conclusion, hair and skin adaptations in granddaughters, arising from dams that experienced in utero heat stress, are documented and include shorter and thicker hair, thinner skin layers, and more numerous but smaller SBG. Similar adaptations have been shown to aid in promoting heat dissipation from the skin's surface in other species, such as pigs and buffalo, and might confer HT_F2_ superior thermal adaptivity in hot climates. Yet, this hypothesis remains to be tested experimentally.

## References

[bib1] Al-Katanani Y.M., Paula-Lopes F.F., Hansen P.J. (2002). Effect of season and exposure to heat stress on oocyte competence in Holstein cows. J. Dairy Sci..

[bib2] Al-Salman A. A.-J., Khairi S.B., Abboud S.S. (2020). Development of skin in cow fetuses at different gestational ages. Biochem. Cell. Arch..

[bib3] Barker D.J.P. (1990). Fetal and infant origins of adult disease. BMJ.

[bib4] Berman A. (2011). *Invited review*: Are adaptations present to support dairy cattle productivity in warm climates?. J. Dairy Sci..

[bib5] Bernabucci U., Lacetera N., Baumgard L.H., Rhoads R.P., Ronchi B., Nardone A. (2010). Metabolic and hormonal acclimation to heat stress in domesticated ruminants. Animal.

[bib6] Braunschweig M., Jagannathan V., Gutzwiller A., Bee G. (2012). Investigations on transgenerational epigenetic response down the male line in F_2_ pigs. PLoS One.

[bib7] Carvalho F.A., Lammoglia M.A., Simoes M.J., Randel R.D. (1995). Breed affects thermoregulation and epithelial morphology in imported and native cattle subjected to heat stress. J. Anim. Sci..

[bib8] Collier R.J., Beede D.K., Thatcher W.W., Israel L.A., Wilcox C.J. (1982). Influences of environment and its modification on dairy animal health and production. J. Dairy Sci..

[bib9] Collier R.J., Gebremedhin K.G. (2015). Thermal biology of domestic animals. Annu. Rev. Anim. Biosci..

[bib10] Dado-Senn B., Field S.L., Davidson B.D., Casarotto L.T., Marrero M.G., Ouellet V., Cunha F., Sacher M.A., Rice C.L., Maunsell F.P., Dahl G.E., Laporta J. (2021). Late-gestation *in utero* heat stress limits dairy heifer early-life growth and organ development. Front. Anim. Sci..

[bib11] Dado-Senn B., Vega Acosta L., Torres Rivera M., Field S.L., Marrero M.G., Davidson B.D., Tao S., Fabris T.F., Ortiz-Colon G., Dahl G.E., Laporta J. (2020). Pre- and postnatal heat stress abatement affects dairy calf thermoregulation and performance. J. Dairy Sci..

[bib12] Davidson B.D., Sarlo Davila K.M., Mateescu R.G., Dahl G.E., Laporta J. (2022). Effect of in utero exposure to hyperthermia on postnatal hair length, skin morphology, and thermoregulatory responses. J. Dairy Sci..

[bib13] Desai M., Hales C.N. (1997). Role of fetal and infant growth in programming metabolism in later life. Biol. Rev. Camb. Philos. Soc..

[bib14] Dikmen S., Hansen P.J. (2009). Is the temperature-humidity index the best indicator of heat stress in lactating dairy cows in a subtropical environment?. J. Dairy Sci..

[bib15] Dikmen S., Khan F.A., Huson H.J., Sonstegard T.S., Moss J.I., Dahl G.E., Hansen P.J. (2014). The SLICK hair locus derived from Senepol cattle confers thermotolerance to intensively managed lactating Holstein cows. J. Dairy Sci..

[bib16] Ebling F., Eady R., Leigh I., Champion R.H., Burton J.L., Ebling F.J.G. (1992). Textbook of Dermatology.

[bib17] Ferreira F.C., Gennari R.S., Dahl G.E., De Vries A. (2016). Economic feasibility of cooling dry cows across the United States. J. Dairy Sci..

[bib18] Hahn G.L. (1999). Dynamic responses of cattle to thermal heat loads. J. Anim. Sci..

[bib19] Hales C.N., Barker D.J.P. (1992). Type 2 (non-insulin-dependent diabetes mellitus: The thrifty phenotype hypothesis. Diabetologia.

[bib20] Hedhly A., Nestorova A., Herrmann A., Grossniklaus U. (2020). Acute heat stress during stamen development affects both the germline and sporophytic lineages in *Arabidopsis thaliana* (L.) Heynh. Environ. Exp. Bot..

[bib21] Houston B.J., Nixon B., Martin J.H., De Iuliis G.N., Trigg N.A., Bromfield E.G., McEwan K.E., Aitken R.J. (2018). Heat exposure induces oxidative stress and DNA damage in the male germline. Biol. Reprod..

[bib22] Laporta J., Ferreira F.C., Ouellet V., Dado-Senn B., Almeida A.K., De Vries A., Dahl G.E. (2020). Late-gestation heat stress impairs daughter and granddaughter lifetime performance. J. Dairy Sci..

[bib23] Latham K.E. (2016). Stress signaling in mammalian oocytes and embryos: A basis for intervention and improvement of outcomes. Cell Tissue Res..

[bib24] Lucas A. (1991). Programming by early nutrition in man. Ciba Found. Symp..

[bib25] Luna E., Bruce T.J.A., Roberts M.R., Flors V., Ton J. (2012). Next-generation systemic acquired resistance. Plant Physiol..

[bib26] Lyne A.G., Heideman M.J. (1959). The pre-natal development of skin and hair in cattle (*Bos taurus* L.). Aust. J. Biol. Sci..

[bib27] McMillen I.C., Robinson J.S. (2005). Developmental origins of the metabolic syndrome: Predictions, plasticity, and programming. Physiol. Rev..

[bib28] Monteiro A.P.A., Tao S., Thompson I.M., Dahl G.E. (2014). Effect of heat stress during late gestation on immune function and growth performance of calves: Isolation of altered colostral and calf factors. J. Dairy Sci..

[bib29] Moyo D., Gomes M., Erlwanger K.H. (2018). Comparison of the histology of the skin of the Windsnyer, Kolbroek, and Large White pigs. J. S. Afr. Vet. Assoc..

[bib30] NRC (1971).

[bib31] Olson T.A., Lucena C., Chase C.C., Hammond A.C. (2003). Evidence of a major gene influencing hair length and heat tolerance in *Bos taurus* cattle. J. Anim. Sci..

[bib32] Porter A.M.W. (1993). Sweat and thermoregulation in hominids. Comments prompted by the publications of P.E. Wheeler 1984–1993. J. Hum. Evol..

[bib33] Porter A.M.W. (2001). Why do we have apocrine and sebaceous glands?. J. R. Soc. Med..

[bib34] Rasmann S., De Vos M., Casteel C.L., Tian D., Halitschke R., Sun J.Y., Agrawal A.A., Felton G.W., Jander G. (2012). Herbivory in the previous generation primes plants for enhanced insect resistance. Plant Physiol..

[bib35] Rocha A., Randel R.D., Broussard J.R., Lim J.M., Blair R.M., Roussel J.D., Godke R.A., Hansel W. (1998). High environmental temperature and humidity decrease oocyte quality in *Bos taurus* but not in *Bos indicus* cows. Theriogenology.

[bib36] Roth Z., Hansen P.J. (2004). Involvement of apoptosis in disruption of developmental competence of bovine oocytes by heat shock during maturation. Biol. Reprod..

[bib37] Rutledge J.J., Monson R.L., Northey D.L., Leibfried-Rutledge M.L. (1999). Seasonality of cattle embryo production in temperate region. Theriogenology.

[bib38] Saravanakumar V.R., Thiagarajan M. (1992). Comparison of sweat glands, skin characters and heat tolerance coefficients amongst Murrah, Surti and non-descript buffaloes. Indian J. Anim. Sci..

[bib39] Sarlo Davila K.M., Hamblen H., Hansen P.J., Dikmen S., Oltenacu P.A., Mateescu R.G. (2019). Genetic parameters for hair characteristics and core body temperature in a multibreed Brahman-Angus herd. J. Anim. Sci..

[bib40] Saxena S.K., Parekh H.K.B., Malik M.R. (1994). Physiological adaptation of sweat glands in crossbred cattle. Indian J. Anim. Sci..

[bib41] Schisa J.A. (2019). Germ cell responses to stress: The role of RNP granules. Front. Cell Dev. Biol..

[bib42] Sikkink K.L., Ituarte C.M., Reynolds R.M., Cresko W.A., Phillips P.C. (2014). The transgenerational effects of heat stress in the nematode *Caenorhabditis remanei* are negative and rapidly eliminated under direct selection for increased stress resistance in larvae. Genomics.

[bib43] Singh A.K., Upadhyay R.C., Malakar D., Singh S.V., Kumar S., Devi R., Singh S.V., Upadhyay R.C., Sirohi S., Singh A.K. (2013). Chapter 6 in Climate Resilient Livestock and Production Systems.

[bib44] Tao S., Monteiro A.P.A., Thompson I.M., Hayen M.J., Dahl G.E. (2012). Effect of late-gestation maternal heat stress on growth and immune function of dairy calves. J. Dairy Sci..

[bib45] Weller J.I., Ezra E., Gershoni M. (2021). Broad phenotypic impact of the effects of transgenerational heat stress in dairy cattle: A study of four consecutive generations. Genet. Sel. Evol..

[bib46] Yadav N.S., Titov V., Ayemere I., Byeon B., Ilnytskyy Y., Kovalchuk I. (2022). Multigenerational exposure to heat stress induces phenotypic resilience, and genetic and epigenetic variations in *Arbidopsis thaliana* offspring. Front. Plant Sci..

[bib47] Yeates N.T.M. (1955). Photoperiodicity in cattle. I. Seasonal changes in coat character and their importance in heat regulation. Aust. J. Agric. Res..

